# Early detection of breast cancer using total biochemical analysis of peripheral blood components: a preliminary study

**DOI:** 10.1186/s12885-015-1414-7

**Published:** 2015-05-15

**Authors:** Udi Zelig, Eyal Barlev, Omri Bar, Itai Gross, Felix Flomen, Shaul Mordechai, Joseph Kapelushnik, Ilana Nathan, Hanoch Kashtan, Nir Wasserberg, Osnat Madhala-Givon

**Affiliations:** 1 Todos Medical Ltd, 1 HaMada St, Rehovot, 76703 Israel; 2 Department Surgery B, Rabin Medical Center, Beilinson Campus, Petach Tikva, and Sackler Faculty of Medicine, Tel Aviv University, Tel Aviv, Israel; 3 Pediatric Hemato-Oncology Unit, Soroka University Medical Center and Faculty of Medicine, Ben-Gurion University of the Negev, Beer-Sheva, Israel; 4 Department of Physics, Ben Gurion University, Beer-Sheva, Israel; 5 Department Clinical Biochemistry, Faculty of Health Sciences, Ben-Gurion University of the Negev, and Institute of Hematology, Soroka University Medical Center, Beer-Sheva, Israel; 6 Division of General Surgery, Rabin Medical Center, Campus Beilinson, Petach Tikva, and Sackler Faculty of Medicine, Tel Aviv, Israel

**Keywords:** Breast cancer detection, Mononuclear cells, Plasma, Infrared spectroscopy

## Abstract

**Background:**

Most of the blood tests aiming for breast cancer screening rely on quantification of a single or few biomarkers. The aim of this study was to evaluate the feasibility of detecting breast cancer by analyzing the total biochemical composition of plasma as well as peripheral blood mononuclear cells (PBMCs) using infrared spectroscopy.

**Methods:**

Blood was collected from 29 patients with confirmed breast cancer and 30 controls with benign or no breast tumors, undergoing screening for breast cancer. PBMCs and plasma were isolated and dried on a zinc selenide slide and measured under a Fourier transform infrared (FTIR) microscope to obtain their infrared absorption spectra. Differences in the spectra of PBMCs and plasma between the groups were analyzed as well as the specific influence of the relevant pathological characteristics of the cancer patients.

**Results:**

Several bands in the FTIR spectra of both blood components significantly distinguished patients with and without cancer. Employing feature extraction with quadratic discriminant analysis, a sensitivity of ~90 % and a specificity of ~80 % for breast cancer detection was achieved. These results were confirmed by Monte Carlo cross-validation. Further analysis of the cancer group revealed an influence of several clinical parameters, such as the involvement of lymph nodes, on the infrared spectra, with each blood component affected by different parameters.

**Conclusion:**

The present preliminary study suggests that FTIR spectroscopy of PBMCs and plasma is a potentially feasible and efficient tool for the early detection of breast neoplasms. An important application of our study is the distinction between benign lesions (considered as part of the non-cancer group) and malignant tumors thus reducing false positive results at screening. Furthermore, the correlation of specific spectral changes with clinical parameters of cancer patients indicates for possible contribution to diagnosis and prognosis.

## Background

Breast cancer is the most common malignancy in women in the United States and the second leading cause of death by cancer. It is estimated that 235,030 new cases of breast cancer will be diagnosed in the United States in 2014 [1]. Early diagnosis is a significant prognostic factor. The American Cancer Society is recommending annual screening mammograms starting at age 40 [2]. Conventional mammography is known to have a sensitivity of about 66 % and specificity of about 92 % [3]. However, recent studies show that screening with mammography does not reduce mortality, it may lead to a 30 % rate of overdiagnosis and may increase unnecessary surgical procedures and patient anxiety [4, 5]. Furthermore, women with dense breasts, in whom mammography is of limited value and high-risk patients with suspicious mammography findings, usually require additional evaluation with ultrasound or magnetic resonance imaging [6]. This may contribute to the diagnosis in some cases but it may increase recall examinations due to false-positive results in others [7, 8]. Alternative methods such as thermography, transillumination, and positron emission tomography, have not been proven yet to have better sensitivity or specificity than mammography [9].

In the last few decades, researchers have introduced the use of serum tumor markers for cancer screening. However, none of the markers tested has proved suitable for screening the entire population because of low specificity and sensitivity at the early stages of disease [10–12]. To improve these results, attempts have been made to apply combinations of markers [13, 14]. Thus, multi-molecular biochemical analysis could be useful for this purpose.

Fourier transform infrared (FTIR) spectroscopy is a simple, rapid, reagents-free biochemical tool that provides information on the total molecular composition of biological samples [15]. Organic compounds absorb infrared light at an energy (wavenumber) corresponding to the nature of the bonds between its atoms, yielding a unique spectral “fingerprint”. Thus, spectroscopy of a biological sample generates an absorption spectrum of the compounds in that sample, reflecting their molecular structure. FTIR spectroscopy is a powerful analytical biochemical and imaging method however, in a complex samples such as blood components, it is complicated to locate a change in a specific molecule due to the overlapping bands and the plenty of vast molecules which compose biological samples. Yet, FTIR can be widely used for differentiating between two different samples and locate the bands and the possible molecules which may contribute to the spectral differences.

FTIR spectroscopy has been found to be useful for the detection and characterization of a broad variety of cancer cells and tissues [15–17]. A previous study by our group in patients with leukemia identified markers of the disease by FTIR spectroscopy of peripheral blood mononuclear cells (PBMCs) which were then used to monitor the disease during chemotherapy [18]. The method was effective even in cases in which blasts were hardly present in the peripheral blood [18], indicating the overall biological influence of malignancy on PBMCs. In another study, our group demonstrated the potential of FTIR analysis of plasma for the detection of solid tumors, mostly breast, colorectal, and lung. Using advanced algorithms, we identified the patients with cancer out of the whole study population with 93.33 % sensitivity and 90.7 % specificity [19].

Prompted by these findings of the systemic effect of malignancy on the FTIR spectra of PBMCs and plasma, in the present study, we sought to investigate the utility of FTIR spectroscopy for breast cancer screening in conjunction with the gold standard diagnostic methods such as mammography and ultrasound.

## Methods

### Patients

The study was conducted at Rabin Medical Center under local Ethics Committee approval at 2011 and 2012. The study group included 29 patients with confirmed breast cancer and 30 control patients without breast cancer as determined by biopsy and standard mammography examination. The control group included 15 patients without pathological findings and 15 patients with benign neoplasms. The patients were randomly selected from population performing routine breast cancer screening and from population prior surgery. Qualified personnel obtained informed consent from each participant. Exclusion criteria were pregnancy, lactation, or presently undergoing fertility treatment, known active inflammation or infection, past treatment for malignant of benign tumor, any type of active autoimmune disease, and current intake of medications such as steroids. Cancer diagnoses were confirmed by clinical, histological, and pathologic means. Cancers were graded according to the National Cancer Institute classification.

### Blood sample collection and preparation

By preparing PBMCs and plasma samples for FTIR measurements we considered all the possible contaminations and interferences from biochemical materials involved in the sample preparation due to the nature of FTIR as highly sensitive biochemical analytical tool. Thus the samples are needed to be clean from reagents. For each participant, 2 ml of blood were collected from a peripheral vein into EDTA tubes (BD Vacutainer® Tubes, BD Vacutainer, Toronto) using standard phlebotomy procedures. Samples were processed within 2 hours of collection. Some of the patients with cancer underwent lymphoscintigraphy with Tc-99 m-labeled nanocolloidal albumin to detect the sentinel node a few minutes before blood collection, but the possibility of an effect of lymphoscintigraphy on the spectra of the blood components was ruled out using FTIR spectroscopy of pure Tc-99 and plasma spectral comparison. The blood was diluted 1:1 in isotonic saline (0.9 % NaCl solution), applied carefully to a Ficoll 1077 gradient (Sigma Chemical Co., St. Louis, MO) in 15 ml collection tubes, and centrifuged at 400 g for 30 min. To discard platelets and cell debris, we placed 1 ml of the plasma in 1.5 ml tubes which were centrifuged at 6000 g for 10 min. The supernatant was transferred to a new 1.5 ml tube, and 0.8 μl of plasma was deposited on a zinc selenide (ZnSe) slide and air-dried for 1 hour under laminar flow. The dried plasma was then subjected to FTIR microspectroscopy.

PBMCs were obtained using a Histopaque 1077 gradient (Sigma, St. Louis, MO) according to the manufacturer’s protocol. The cells were aspirated from the interface, rinsed twice with isotonic saline at 250 g, and re-suspended in 5 μl fresh isotonic saline. Thereafter, 0.4 μl of washed cells were deposited on ZnSe slides to create an approximate uniform layer of cells. The cells were air-dried for 1 hour under laminar flow and analyzed by FTIR microspectroscopy. The samples need to be dried since water molecules strongly absorb infrared light which may mask the signal from the sample.

### *FTIR* microspectroscopy

All spectroscopy studies were performed with the Nicolet Centaurus FTIR microscope equipped with a liquid-nitrogen-cooled mercury-cadmium-telluride detector coupled to Nicolet iS10 OMNIC software (Nicolet, Madison, WI). To achieve a high signal-to-noise ratio (SNR), 128 co-added scans were collected in each measurement in the 700 to 4000 cm^−1^ wavenumber region. At a spectral resolution of 4 cm^−1^ (0.482 cm^−1^ data spacing), each spectrum contains 6845 data points. The dimensions of the measurement site were 100 μm X 100 μm. Measurements were performed in transmission mode at least 5 times at different spots in each sample of PBMCs or plasma.

### Spectral preprocessing

The FTIR spectra for PBMCs and plasma were first examined for unsuccessful measurements, such as absorption intensity above or below normal (defined as 0.5 to 1 absorption units according to Amide I band) and water vapor contamination. Next, we focused on the relevant region of 1800–700 cm^−1^ which contains most of the biochemical data of PBMCs and plasma. Following standard vector normalization to obtain a unity total energy of each spectrum [19, 20], we applied a moving average filter to increase the SNR. Finally, we sought a numerical estimation for the second derivative of the spectra to accentuate the bands, reduce the background interference, and reveal the genuine biochemical characteristics [21]. Although the second-derivative method is known to be highly susceptible to full width at half maximum changes in the infrared bands, these changes are not relevant in biological samples in which all cells of the same type and plasma are composed of similar basic components that yield relatively broad bands [22]. Spectrum parameters were calculated by our in-house algorithms; the code was employed using MATLAB (Version R2011B: MathWorks Inc., Natick, MA).

### Feature selection

The spectra obtained contained 2282 data points or dimensions. For successful and less complex classification, the number of dimensions needed to be reduced. Our goal was to identify a subset of specific wavenumbers or intervals in the spectra that represented the different spectral patterns of the groups. To improve the model, we defined two criteria for potential feature evaluation. First, we performed a Student’s *t*-test analysis between the no cancer class (benign or no breast tumor) and the cancer class. A feature was considered significant at *P* <0.005. Next, for each potential feature, we obtained the probability distribution of each class and measured the similarity of the probability density functions. In this manner, we were able to evaluate the amount of overlap between the two populations.

### Statistical analysis

Following feature selection, quadratic discriminant analysis (QDA), a multivariate data analysis method, was performed to classify the different groups under the assumption that each feature is normally distributed. The QDA classifier produces a new discriminative score for each subject that can be classified according to the cut-off point. The best cut-off point was determined by creating a receiver operating characteristics (ROC) curve and choosing the one with the best performance [23]. Monte-Carlo cross-validation was used to determine the accuracy of classifier predictions for different cut-offs [23].

## Results

### FTIR- MSP analysis of PBMC spectra

**Fig. 1:**
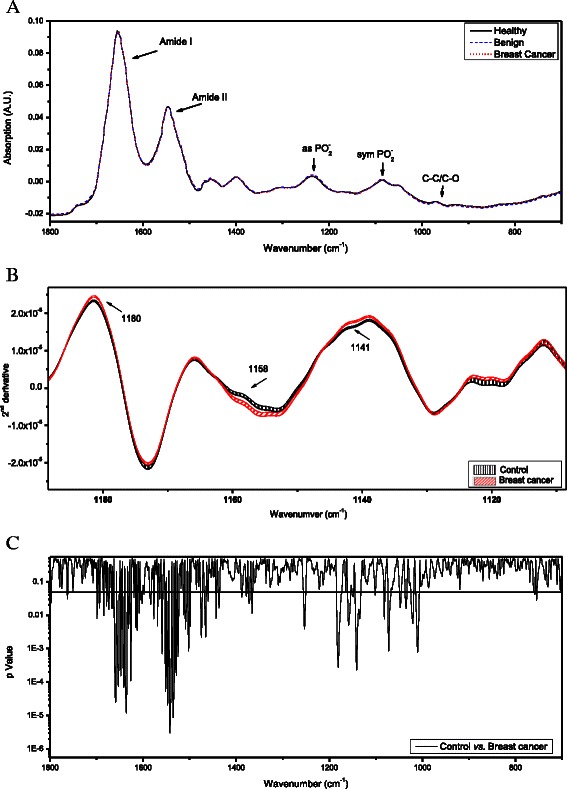
FTIR-MSP spectra of PBMCs of cancer patients and healthy controls. (**a**) Average of the absorption spectra of PBMC of each study group between 1800 cm^−1^ and 700 cm^−1^. The spectra are vector normalized. Each spectrum of a single subject is an average of five measurements at different locations of the PBMCs dried film. The absorbance bands of the major functional bonds of the bio-molecules are marked. (**b**) Second derivative expanded spectra of PBMCs from each subjects’ group are presented. The mean ± SEM for each of the data sets is represented by the thickness of the graph lines. (**c**) *t*-test analysis of the second derivative spectra of control group vs. cancer patients group. The *t*-test is represented by p-Value (in log scale) for each wavenumber along the IR spectra. Statistically significant differences are present at various wave-numbers which are indicated by p-values below 0.05 represented by the black horizontal solid line

The characteristics of the study subjects are shown in Table 1. Using FTIR-MSP, we first characterized the spectral differences among women with malignant breast tumor, benign breast tumor, or no breast tumor. The averages of the infrared spectra of the PBMCs in each group are presented in Fig. 1.

**Table 1:** Demography, clinical characteristics and diagnosis of the control and cancer groups included in this study

	Cancer	Control
No. of patients	29	30
Age		
Range	28.6 – 83.9	20.4 – 75.8
Average ± STD	60.1 ± 13.2	45.7 ± 16.5
History of Smoking	29.6 %	19.2 %
Family History of Cancer		
Breast	41.3 %	63.3 %
Ovary	31 %	53.3 %
Histology		
IDC	(22) 75.9 %	
ILC	(4) 13.8 %	
IDC + ILC	(1) 3.4 %	
Mucinous Ca	(1) 3.4 %	
HG DCIS	(1) 3.4 %	
Stage		
I	(1) 3.4 %	
II	(14) 48.3 %	
III	(4) 13.8 %	
NA/NR	(10) 34.5 %	
Nodule Size (mm)		
≤10	(8) 27.6 %	
10 ≤ 20	(8) 27.6 %	
20<	(11) 37.9 %	
NA/NR	(2) 6.9 %	
Receptors		
ER+	(25) 86.2 %	
PR+	(22) 75.9 %	
Her2+	(2) 6.9 %	
NA/NR	(4) 13.8 %

Figure 1a shows the macromolecules composing the PBMC spectrum. The 1800–1500 cm^−1^ (amide I and amide II) region contains mostly information on protein content and secondary structure. The 1300–800 cm^−1^ region is due to vibrations of functional groups such as PO_2_^−^, CO and CC present in proteins, lipids, nucleic acids, and carbohydrates [24, 25]. It was difficult to distinguish among the three study groups on the basis of the raw infrared absorption spectra, and further analysis was needed.

Figure 1b shows an expanded region of the spectra resulting from applying a second derivative to the original absorption spectra of the PBMCs. The thickness of the lines represents the standard error of the mean (SEM). The second derivative is a common mathematical operation on the IR spectra which reveals the bands composed within the broad main absorption bands. Each band in the absorption spectra is represented as sharper and more pronounced minima in the second derivative spectra.

Statistical analysis of the second derivative spectra revealed significant differences mainly between the patients with malignancy and the patients without malignancy (namely subjects without tumors and patients with benign tumors). Specifically, in the PBMCs from patients with malignancy, a decline in absorption (higher value in the second derivative) was found at ~1140 cm^−1^ which corresponds to the oligosaccharide C-OH stretching band [25]. In addition, a morphological change was observed at the amide II region at ~1545 cm^−1^.

Since there are no significant clinical differences between patients without tumors and patients with benign tumors [26], they were combined into a single control group for all further comparisons and statistical analyses.

To statistically identify which region of the infrared spectra was abnormal in the patients with malignancy, we applied a *t*-test analysis to all second derivative spectra. The results are presented in Fig. 1c. Comparison of the PBMCs from the cancer and control groups revealed two main regions with a significant difference (*P* <0.05): 1700–1450 cm^−1^, which is due to amide I and amide II absorption, and 1180–1000 cm^−1^, which is mainly due to symmetric PO_2_^−^ stretching, C-C symmetric vibrations, and C-O symmetric vibrations of proteins, nucleic acids, carbohydrates, and phospholipids.

**Fig. 2:**
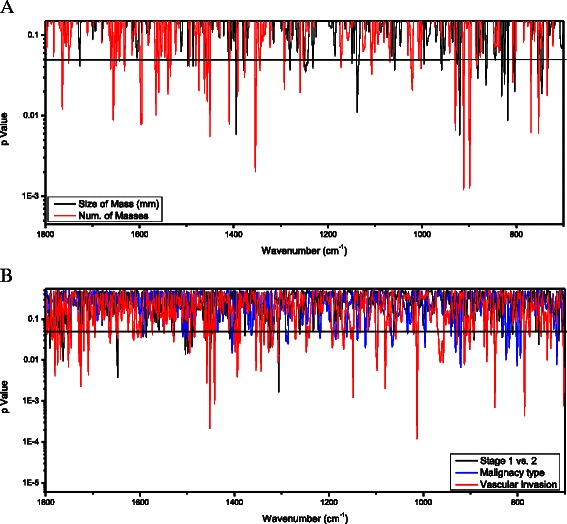
*T*-test analysis of the FTIR-MSP second derivative spectra of PBMCs of cancer patients group. The *t*-test is represented by p-Value (in log scale) for each wavenumber along the IR spectra. Comparison between the following pathological parameters: (**a**) Size of mass bellow 20 mm vs. above 20 mm; Single mass vs. multiple masses; (**b**) Cancer stage 1 vs. stage 2; malignancy type - Ductal vs. Lobular Carcinoma; positive vs. negative for vascular invasion. Statistically significant differences are present at various wavenumbers which are indicated by p-values below 0.05 marked by black horizontal solid line

To further understand the influence of cancer on PBMC biochemistry, the spectral results were analyzed by the clinical parameters within the group of patients with malignancy. The results are presented in Fig. 2. Figure 2a shows that analysis by mass size (solid line) yielded a significant difference in absorption at several wavenumbers, such as 1394 cm^−1^ (*P* = 0.0058), 1137 cm^−1^ (*P* = 0.011), and 920 cm^−1^ (*P* = 0.0057), between patients with a malignant mass of less or more than 20 mm. Number of masses (one vs. two or more; dotted line) had an even greater effect on absorption: at 1353 cm^−1^ (*P* = 0.002), 911 cm^−1^ (*P* = 0.0012), and 899 cm^−1^ (*P* = 0.0013). On analysis by lymph node involvement (data not shown), most of the changes in absorption were located at ~1400 cm^−1^ and ~800 cm^−1^. As shown in Fig. 2b, cancer stage (1 or 2; solid line) had no significant effect on absorption except at 1306 cm^−1^ and 1647 cm^−1^. Type of cancer (invasive ductal carcinoma or lobular carcinoma; dotted line), affected the PBMC spectra mainly at ~920 cm^−1^ and ~801 cm^−1^ and, at a lower level of significance, at ~1404 cm^−1^ and ~1120 cm^−1^. Vascular involvement (dashed line) had a highly significant effect on absorption along multiple regions of the spectra, mainly at 1012 cm^−1^ (*P* = 0.00012) and 1452 cm^−1^ (*P* = 0.00022).

### FTIR-microscopy analysis of plasma

**Fig. 3:**
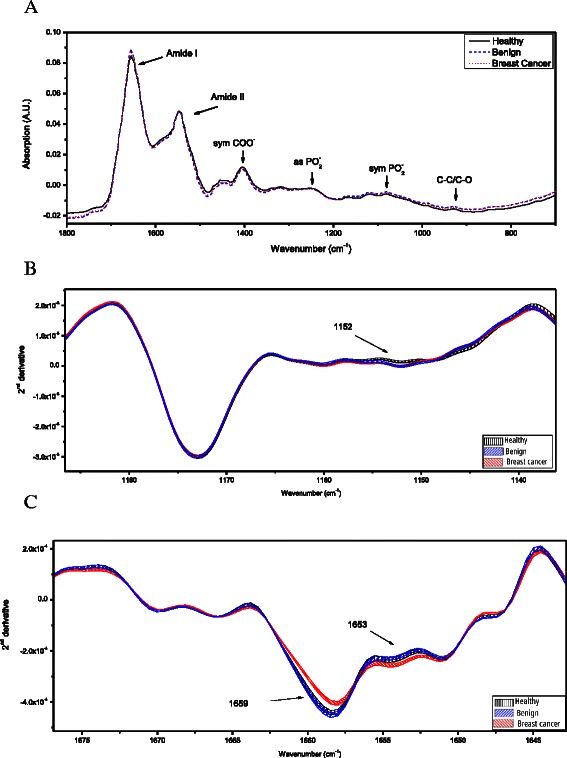
FTIR-MSP spectra of plasma of cancer patients, ‘benign’ patients and healthy controls. (**a**) Average of the absorption spectra of plasma of each study group between 1800 cm^−1^ and 700 cm^−1^. The spectra are vector normalized. Each spectrum of a single subject is an average of five measurements at different locations of the plasma dried film. The absorbance bands of the major functional bonds of the bio-molecules are marked. (**b**) and (**c**), Expanded second derivative spectra of plasma from each subjects’ group are presented. The mean ± standard error of the mean (SEM) for each data set (healthy, benign, and cancer) is represented by the thickness of the curves

Figure 3 presents the averages of the infrared spectra of the dried plasma for each group. As shown in Fig. 3a, the pattern was much different from that of the PBMC, mainly because of the relatively high content of proteins (absorption band at ~1400 cm^−1^ due to COO^−^ and symmetric CH_3_ bending of methyl groups) rather than nucleic acids (absorption band at ~1240 cm^−1^ and ~1080 cm^−1^ due to PO_2_^−^) [27].

There were clear differences in the absorption spectra of plasma derived from the patients with malignant tumors, patients with benign tumors, and subjects without tumor. To gain more information and to reduce the influence of scattering, we analyzed the second-derivative spectra. The results are presented in Fig. 3b and c. Significant differences (beyond SEM) were found at ~1160 cm^−1^ (corresponding to absorbance of C-O of proteins and carbohydrates) and at ~1655 cm^−1^ (corresponding to absorbance of amide I) [25, 27]. A common spectral trend was observed for patients with malignant or benign tumor at ~1160 cm^−1^. Plasma from both tumor groups showed significantly higher absorption at 1152 cm^−1^ than plasma from the healthy subjects. Interestingly, in the amide I region, the spectra of the benign group were more similar to the spectra of the healthy group than to the spectra of the malignancy group (Fig. 3c), compatible to the PBMC results.

**Fig. 4:**
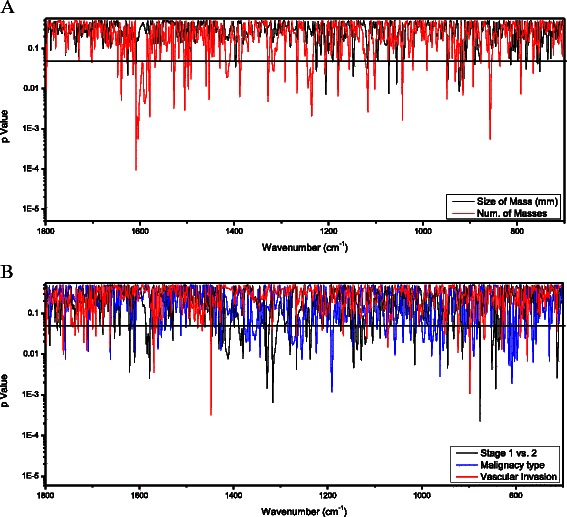
*T*-test analysis of the FTIR-MSP second derivative spectra of plasma of cancer patients group. The *t*-test is represented by p-Value (in log scale) for each wavenumber along the IR spectra. Comparison between the following pathological parameters: (**a**) Size of mass bellow 20 mm vs. above 20 mm; Single mass vs. multiple masses (**b**) Cancer stage 1 vs. stage 2; malignancy type - Ductal vs. Lobular Carcinoma; positive vs. negative for vascular invasion. Statistically significant differences are found at various wave-numbers indicated by p-values below 0.05 displayed by the black horizontal solid line

Changes in plasma biochemical composition by clinical parameters within the group of cancer patients are presented in Fig. 4. Figure 4a shows significant differences in absorption bands at three main regions between patients with a malignant mass larger or smaller than 20 mm (solid line): 923 cm^−1^, 1072 cm^−1^ and 1205 cm^−1^. More significant biochemical changes were observed on analysis by number of tumor masses (one vs. two or more). For most of the bands, the *P* value was below 0.01; the most prominent bands were found at 1608 cm^−1^, due to COO_2_^−^ polysaccharides and adenine vibration in DNA, and at 857 cm^−1^ due to C3’ endo/anti (α-form helix) conformation [25]. By contrast, lymph node involvement was not associated with any significant change in absorption (data not shown). Analysis by tumor stage (1 or 2, solid line; Fig. 4b) yielded significant differences mainly at ~1316 cm^−1^ (amide III) and around 876 cm^−1^ (C3’ endo/anti α-form helix), and by tumor type (ductal or lobular carcinoma, dotted line), mainly at ~1190 cm^−1^, 961 cm^−1^ and ~808 cm^−1^ which correspond to deoxyribose, C-O deoxyribose, C-C, and C3’ endo/anti (α-form helix) conformation, respectively [25]. Vascular involvement (dashed line) had a highly significant effect on only two regions of the spectra: 1447 cm^−1^ and 898 cm^−1^.

**Fig. 5:**
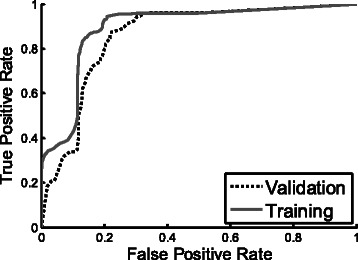
ROC curves for healthy and benign vs. cancer. ROC curves were calculated using combined features selected from PBMCs and Plasma spectral data of each subject. Training set ROC (solid curve) as well as validation set ROC (dot curve) used in the Monte-Carlo Cross validation is presented

To determine if our method is suitable for the detection of cancer and to make use of all the available biochemical information on each patient, we combined the spectral data of the PBMC and plasma for 26 controls (patients with benign tumors + healthy controls) and 24 subjects with cancer. (A few plasma samples were excluded because of hemolysis). Our mathematical model generated a QDA score for each subject and a ROC curve for determining its sensitivity and specificity for identifying patients with cancer (Fig. 5). The training set curve in the figure appears in a solid line, and the validation set curve, in dashed line. The area under the curve was 0.898 [SD: 0.894 - 0.903] and 0.857 [SD: 0.835 - 0.878] for the training and validation sets respectively, indicating good accuracy for the diagnostic test by the traditional academic system. Using the ROC curve, we were able to select the optimal cut-off that distinguished the two groups. This yielded a sensitivity of 89 % and a specificity of 80 % for the training set. The validation values were similar: 87 % and 78 %, respectively.

## Discussion

The present study describes a novel concept for breast cancer detection based on the immune system response to the presence of tumor in the body rather than on observation of the tumor cells themselves. Furthermore, by using infrared spectroscopy, we were able to analyze the entire biochemical signature (including proteins, lipids, nucleic acids, and carbohydrates) of the affected immune cells rather than focusing on a single specific protein as a biomarker. We also analyzed the malignancy-induced biochemical changes in plasma to obtain more information about the disease and as an auxiliary means of cancer detection.

The results provide evidence that the PBMCs and plasma of patients with breast cancer are biochemically distinct from the PBMCs and plasma of healthy subjects, including patients with benign tumors, with no significant differences in PBMC spectra between patients with benign tumors and healthy subjects. For plasma, there was a biochemical similarity between patients with benign tumors and healthy subjects for some spectral absorption bands, and between patients with benign tumors and patients with malignant tumors for other absorption bands. Further analysis of the data within the group of cancer patients revealed a correlation of the spectral changes of PBMCs and plasma with clinically relevant parameters known to influence the diagnosis and prognosis of breast cancer, such as disease stage and vascular invasion.

Previous studies of cancer cells and tissues using FTIR spectroscopy reported an abnormal biochemical profile, expressed by various changes in the phosphate region which corresponds mainly to nucleic acids and carbohydrates [28, 29]. Others also noted a significant increase in the ratio of CH_2_/CH_3_ in the higher region of lipids and protein absorption [29, 30]. These changes were consistent for most of the tumors and depended on the stage of disease [28, 30]. They were compatible with our findings in an earlier study of PBMC biochemistry in patients with acute leukemia [18]. However, in the present study, which included patients with solid tumors, there was no significant change in CH_2_/CH_3_. The major changes observed between the groups were found in proteins structure and in several functional groups of nucleic acids, carbohydrates and phospholipids, suggesting that PBMCs from patients with solid tumors have a different profile than PBMCs from patients with hematological malignancies. Thus, our results indicate cancer-type-dependent changes in the PBMC population.

The differences in PBMC biochemistry between patients with and without cancer may be related to malignancy-induced biological effects, such as changes in the composition of the mononuclear population; specifically, the relationships between B and T cells [31, 32]. The presence of CD4 + CD25- T cells in the peripheral blood as well as in the tumor site leads to a significant increase in the number of regulatory T cells (Treg cells, CD4 + CD25+) [33, 34]. These findings have been reported not only in breast cancer [31, 35, 36], but also in gastrointestinal [37], and lung cancer [38]. Treg cells regulate effector T cells and disable them in order to prevent them from attacking the tumor [33]. The level of Treg cells is apparently correlated with disease stage and declines with tumor dissection [11, 37, 38]. Studies have also provided evidence of the role of natural killer cells as a prognostic parameter and therapy target [39–41]. These studies support our finding of the contribution of clinical parameters (tumor size, blood vessel and lymph node involvement) to the biochemical changes in PBMCs and highlight the potential of FTIR-spectroscopy as a prognostic and treatment follow-up tool. Although the changes in the PBMC population may be correlated with stage of disease, in the present study, there were no cases of advanced-stage breast cancer, so further studies in animal and human models are needed to address this issue.

Many studies have investigated the difference between healthy and malignant tumors, but only a few addressed the biochemistry of benign tumors (45, 46). They found no or only slightly significant differences from malignant tumors [42, 43]. On the contrary, in the present study, only small differences were observed in the PBMC spectra between patients with benign tumors and healthy subjects. However, more extensive studies are needed to verify these preliminary results.

Our previous study showed that FTIR spectroscopy of plasma is a promising mean for distinguishing patients with cancer from healthy subjects however the benign tumors were not investigated by Ostrovsky et al. [19]. Most of the common serum biomarkers cannot be used for distinguishing between benign and malignant tumors [42, 44], perhaps because of the immunological similarity of the tissues. Indeed, in the present study, we identified several vibrational bands in the plasma spectra that were common to both benign and malignant tumors which correspond to carbohydrates and proteins. We further identified bands which are common to healthy and benign groups in the Amide I band which correspond mainly to protein secondary structure. Thus, the significant contribution to cancer detection may be related to the structure of proteins in the plasma rather than carbohydrates. For our purposes, we can relate only to the bands that are common to benign and healthy tissues and improve the detection of malignant tumors.

The algorithm presented here makes use of the global biochemical information obtained both for PBMCs and plasma. The sensitivity was about 90 % and the specificity was about 80 %. These values are promising considering that we were able to distinguish between nonmalignant and malignant tumors and most of the patients with malignancy were at early stages of the disease. We aim to further improve our algorithm with a larger sample size.

## Conclusion

In light of the present preliminary results, we conclude that analysis of the biochemical composition of the PBMC and plasma using FTIR spectroscopy may serve as a simple, cost effective, automated and minimally invasive test for the presence of breast cancer. Additional studies to improve and validate our results are required before this method can be applied to clinical practice, in conjunction with other accepted diagnostic methods such as mammography. Expansion of this preliminary study will provide further insight into the full potential of FTIR spectroscopy for mass screening and early detection of breast cancer.

## Abbreviations

AUC: Area under the curve
EDTA: Ethylenediaminetetraacetic acid
FTIR: Fourier transform infrared
PBMCs: Peripheral blood mononuclear cells
QDA: Quadratic discriminant analysis
ROC: Receiver operating characteristics
SNR: Signal-to-noise ratio
ZnSe: Zinc selenide
IDC: Invasive ductal carcinoma
ILC: Invasive lobular carcinoma
HG DCIS: high-grade dysplasia/carcinoma in situ
ER: estrogen receptor
PR: progesterone receptor
NA: not applicable
NR: not relevant
